# Supplementary material for: Opposite Roles of BAP1 in Overall Survival of Uveal Melanoma and Cutaneous Melanoma
Source: J Clin Med. 2020 Feb 3;9(2):411. doi: 10.3390/jcm9020411 (PMC7074098; doi:10.3390/jcm9020411)
Supplement: Supplementary file 1 [file jcm-09-00411-s001.pdf]

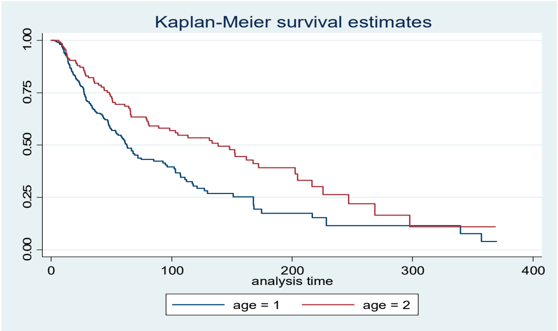

Figure S1. Age as a significant predictor for overall survival in CM. age = 1, >50; Age = 2, <= 50.

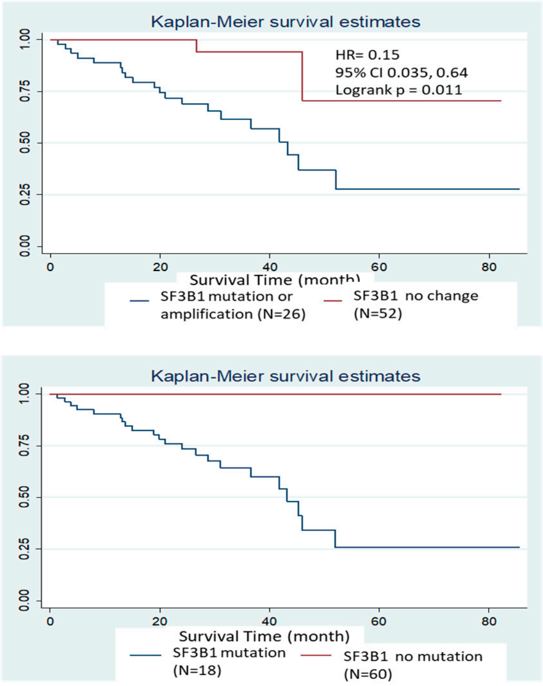

Figure S2. role of SF3B1 in UM melanoma survival.

Table S1. Mean BAP1 mRNA levels in UM and CM are correlated with copy number of BAP1 gene.

Tumor	BAP1 CNV	N	Mean	Std. Err	*p* value
UM	No alteration	36	4750.5	253.7	p <0.0001
	Hemizygous deletion	44	1394.9	169.4	
CM	No alteration	227	2849.4	60.4	p<0.0001
	Hemizygous deletion	74	1977.5	100.6	
	Amplification	66	3540.2	154.7	

Table S2. BAP1 amplification predicts better overall survival comparing to diploids.

	HR	95% CI		p_BAP1	Additional Variable
**Model 1**	0.59	0.39	0.90	0.013	Age in 4 category*
Model 2	0.68	0.44	1.05	0.080	Stage (T1-T4 stages)

Table S3. Cox survival analysis assuming 84.5 month follow-up time in CM patients

	HR	95% CI		p_BAP1
**BAP1**	0.73	0.54	0.98	0.041

Table S4. Shared genes co-expressed with BAP1 in UM and CM.

			UM			CM		
**Num**	**gene**	**cytoband**	**Coef**	**pvalue**	**qvalue**	**Coef**	**pvalue**	**qvalue**
1	ABCF3	3q27.1	0.654	4.70E-11	2.60E-09	0.507	3.10E-32	1.70E-29
2	ABHD3	18q11.2	-0.647	9.30E-11	4.50E-09	-0.426	2.90E-22	3.10E-20
3	AKAP5	14q23.3	-0.577	2.10E-08	4.40E-07	-0.427	2.70E-22	2.90E-20
4	ALG3	3q27.1	0.574	2.50E-08	5.20E-07	0.460	3.80E-26	8.20E-24
5	ATG2A	11q13.1	0.523	6.30E-07	8.30E-06	0.396	3.60E-19	1.90E-17
6	ATP2B1	12q21.33	-0.548	1.50E-07	2.40E-06	-0.468	4.20E-27	1.10E-24
7	BAG6	6p21.33	0.567	4.30E-08	8.30E-07	0.423	7.00E-22	7.10E-20
8	CERKL	2q31.3	-0.513	1.20E-06	0.000014	-0.432	7.80E-23	9.80E-21
9	CGAS	6q13	-0.519	8.20E-07	0.00001	-0.427	2.50E-22	2.70E-20
10	CUTA	6p21.32	0.655	4.20E-11	2.30E-09	0.408	2.20E-20	1.50E-18
11	DVL3	3q27.1	0.770	6.50E-17	3.50E-14	0.451	4.80E-25	8.80E-23
12	EEFSEC	3q21.3	0.764	1.70E-16	7.80E-14	0.411	1.10E-20	7.90E-19
13	FAM131A	3q27.1	0.712	1.40E-13	1.90E-11	0.429	1.30E-22	1.60E-20
14	FGL2	7q11.23	-0.546	1.70E-07	2.60E-06	-0.415	4.00E-21	3.30E-19
15	HMG20B	19p13.3	0.565	4.60E-08	8.80E-07	0.429	1.60E-22	1.80E-20
16	ITGA4	2q31.3	-0.511	1.30E-06	0.000015	-0.455	1.80E-25	3.50E-23
17	KCTD12	13q22.3	-0.512	1.20E-06	0.000014	-0.428	1.70E-22	1.90E-20
18	NCBP2AS2	3q29	0.809	1.10E-19	2.60E-16	0.438	1.50E-23	2.10E-21
19	NUCB2	11p15.1	-0.516	9.50E-07	0.000012	-0.487	1.80E-29	6.90E-27
20	NUP88	17p13.2	-0.574	2.50E-08	5.20E-07	-0.399	1.80E-19	1.00E-17
21	PMEL	12q13.2	0.618	9.80E-10	3.10E-08	0.430	1.00E-22	1.20E-20
22	PPP2R5C	14q32.31	-0.562	5.80E-08	1.10E-06	-0.407	3.30E-20	2.20E-18
23	RABEP1	17p13.2	-0.511	1.20E-06	0.000015	-0.410	1.60E-20	1.20E-18
24	RAP1B	12q15	-0.559	6.90E-08	1.20E-06	-0.491	5.80E-30	2.50E-27
25	RBMS1	2q24.2	-0.543	1.90E-07	2.90E-06	-0.442	5.40E-24	8.20E-22
26	RIDA	8q22.2	-0.573	2.90E-08	5.80E-07	-0.402	8.40E-20	5.10E-18
27	RRM2B	8q22.3	-0.629	4.10E-10	1.50E-08	-0.422	8.20E-22	8.20E-20
28	RTL8A	Xq26.3	0.558	7.50E-08	1.30E-06	0.415	4.20E-21	3.40E-19
29	SCFD1	14q12	-0.511	1.30E-06	0.000015	-0.402	9.80E-20	5.90E-18
30	SEPTIN7	7p14.2	-0.548	1.40E-07	2.30E-06	-0.408	2.60E-20	1.70E-18
31	SLC6A8	Xq28	0.577	2.10E-08	4.50E-07	0.404	6.10E-20	3.90E-18
32	TMEM250	9q34.3	0.557	8.30E-08	1.50E-06	0.413	7.00E-21	5.40E-19
33	TMEM87B	2q13	-0.603	3.20E-09	8.80E-08	-0.402	9.30E-20	5.60E-18
34	TMX1	14q22.1	-0.524	6.00E-07	7.90E-06	-0.409	2.00E-20	1.40E-18
35	TRAM1	8q13.3	-0.547	1.60E-07	2.50E-06	-0.406	3.70E-20	2.50E-18
36	UBE2V2	8q11.21	-0.622	7.40E-10	2.50E-08	-0.396	3.80E-19	2.00E-17
37	UBE2W	8q21.11	-0.515	1.00E-06	0.000012	-0.400	1.30E-19	7.60E-18

38	ZEB1	10p11.22	-0.589	9.40E-09	2.20E-07	-0.472	1.70E-27	4.70E-25
39	ZNF687	1q21.3	0.564	4.90E-08	9.30E-07	0.424	4.60E-22	4.80E-20

Table S5. The top ranked (by Spearson's coefficient) BAP1 co-expressed genes in UM and CM.

Tumor	Gene	Cytoband	Coeff	p-Value	q-Value
UM	ZBTB11-AS1	3q12.3	0.831	1.41E-21	2.74E-17
UM	SLC41A3	3q21.2-q21.3	0.813	5.51E-20	1.79E-16
UM	NCBP2AS2	3q29	0.809	1.15E-19	2.58E-16
UM	HMCES	3q21.3	0.802	4.16E-19	6.23E-16
UM	HTR2B	2q37.1	-0.791	2.41E-18	2.61E-15
UM	IFT122	3q21.3-q22.1	0.785	6.74E-18	6.57E-15
UM	LIMS2	2q14.3	0.782	1.08E-17	9.60E-15
UM	FBXO17	19q13.2	0.781	1.33E-17	1.04E-14
UM	MCM2	3q21.3	0.779	1.90E-17	1.43E-14
UM	ACSF2	17q21.33	0.778	2.11E-17	1.52E-14
UM	PCBD2	5q31.1	-0.777	2.35E-17	1.63E-14
UM	GTF2H4	6p21.33	0.773	4.14E-17	2.60E-14
UM	PHYHD1	9q34.11	0.772	4.85E-17	2.95E-14
UM	SCGB1B2P	19q13.11	0.772	5.15E-17	3.04E-14
UM	PXDC1	6p25.2	0.771	6.43E-17	3.50E-14
UM	DVL3	3q27.1	0.770	6.47E-17	3.50E-14
UM	PPM1K	4q22.1	-0.769	8.35E-17	4.02E-14
UM	TFAP2A	6p24.3	0.765	1.42E-16	6.57E-14
UM	EEFSEC	3q21.3	0.764	1.74E-16	7.76E-14
UM	GPR153	1p36.31	0.763	1.99E-16	8.60E-14
CM	YAF2	12q12	-0.547	2.8994E-38	2.436E-35
CM	C12ORF4	12p13.32	-0.530	1.3194E-35	9.5019E-33
CM	UBXN4	2q21.3	-0.521	3.2388E-34	2.1067E-31
CM	ABCF3	3q27.1	0.507	3.0931E-32	1.6856E-29
CM	PPP1R12A	12q21.2	-0.506	4.2731E-32	2.2674E-29
CM	FAS	10q23.31	-0.500	3.6746E-31	1.8998E-28
CM	TANK	2q24.2	-0.496	1.0685E-30	5.2551E-28
CM	USP15	12q14.1	-0.495	1.343E-30	6.2145E-28
CM	GPATCH3	1p36.11	0.491	5.5087E-30	2.4147E-27
CM	RAP1B	12q15	-0.491	5.7935E-30	2.4855E-27
CM	CAB39	2q37.1	-0.489	9.4353E-30	3.9636E-27
CM	RPAP3	12q13.11	-0.489	9.8421E-30	4.0501E-27
CM	THAP1	8p11.21	-0.489	1.1056E-29	4.4587E-27
CM	TOP1P1	1q24.3	-0.488	1.1658E-29	4.5208E-27
CM	NUCB2	11p15.1	-0.487	1.8459E-29	6.8926E-27
CM	JAK2	9p24.1	-0.484	4.7925E-29	1.757E-26
CM	SCAF11	12q12	-0.482	8.2342E-29	2.9649E-26
CM	ST3GAL3	1p34.1	0.481	1.0297E-28	3.5799E-26
CM	PEX10	1p36.32	0.481	1.0483E-28	3.5826E-26
CM	ZNF362	1p35.1	0.479	1.7553E-28	5.8991E-26

Table S6. Over-expressed and under-expressed proteins in BAP1 mRNA high CM tumors.

Gene	Cytoband	p-Value	q-Value	Tendency
KIT	4q12	7.58E-08	7.8E-06	Over-expressed
CDH1	16q22.1	0.00004141	0.001706	Over-expressed
CDK1	10q21.2	0.000184	0.00554	Over-expressed
BCL2	18q21.33	0.0003191	0.007422	Over-expressed
CTNNB1	3p22.1	0.0003243	0.007422	Over-expressed
EIF4EBP1_PT70	17q21	0.0006336	0.0119	Over-expressed
PRKCA_PS657	4q24	1.81E-08	3.72E-06	Under-expressed
PRKCA	17q24.2	0.000001366	9.38E-05	Under-expressed
IGFBP2	2q35	0.000003321	0.000171	Under-expressed
PREX1	20q13.13	0.0001883	0.00554	Under-expressed
CASP7	10q25.3	0.0005957	0.0119	Under-expressed
LCK	1p35.2	0.0008538	0.0147	Under-expressed
MAPK9	5q35.3	0.001373	0.0217	Under-expressed
TGM2	20q11.23	0.001531	0.0225	Under-expressed
PRKCB_PS660	5q21-q22	0.003053	0.0419	Under-expressed
